# *Serpentirhabdias mexicanus* n. sp. (Nematoda: Rhabdiasidae), a parasitic lungworm of the nauyaca viper *Bothrops asper* (Serpentes: Viperidae) in the Mexican Neotropics

**DOI:** 10.1007/s11230-023-10144-x

**Published:** 2024-02-06

**Authors:** Andrés Velázquez-Brito, Luis García-Prieto, Uriel Garduño-Montes de Oca, Víctor Manuel Sosa-Jiménez, Mirna Crizel Vera-Chávez, Virginia León-Règagnon

**Affiliations:** 1 Laboratorio de Helmintología, Instituto de Biología, Universidad Nacional Autónoma de México, Coyoacán, 04510 Mexico, Mexico; 2 Laboratorio de Herpetología, Instituto de Biología, Universidad Nacional Autónoma de México, Coyoacán, 04510 Mexico, Mexico; 3 Posgrado en Ciencias Biológicas, Universidad Nacional Autónoma de México, Apartado 70-153, CP 04510 Mexico, Mexico

## Abstract

*Serpentirhabdias mexicanus* **n. sp.** (Nematoda: Rhabdiasidae) is described from the lung of the nauyaca viper *Bothrops asper* in Puebla State, central Mexico. This new species is the fifth of the genus described having onchia. Among the species included in this group, the new species is morphologically closest to *S. viperidicus* and *S. atroxi*. However, it differs from both species mainly by having only one excretory gland (compared to two present in *S*. *viperidicus* and *S*. *atroxi*). In addition, *S. mexicanus* **n. sp.** can be separated of *S*. *viperidicus* by tail length, shape of vulval lips, geographic distribution and host species and from *S. atroxi* by body length, number of papillae in the cephalic region, as well as the host species and geographic distribution. In the present study, we propose the new species based on morphological, host spectrum and genetic evidence. Phylogenetic analysis indicated *Serpentirhabdias* as a monophyletic group, with two subgroups that are congruent with the presence/absence of onchia in the esophagostome, host association and other relevant morphological characters.

## Introduction

Rhabdiasidae Railliet, 1915, a family of nematodes parasitic mainly of lungs of amphibian and reptiles, comprises eight genera: *Acanthorhabdias* Pereira, 1927, *Chabirenia* Lhermitte-Vallarino, Bain, Deharo, Bertani, Voza, Attout & Gaucher, 2005, *Entomelas* Travassos, 1930, *Kurilonema* Szczerbak & Sharpilo, 1969, *Neoentomelas* Hasegawa, 1989, *Pneumonema* Johnston, 1916, *Rhabdias* Stiles & Hassall, 1905 and *Serpentirhabdias* Tkach, Kuzmin & Snyder, 2014 (Tkach et al., 2014). Currently, *Serpentirhabdias* includes twenty valid species (Kuzmin and Tkach, 2022)**.** Eleven of these have been registered in the Americas, parasitising members of Serpentes; particularly from Viperidae, only two species are known: *S. viperidicus* Morais et al., 2017 and *S. atroxi* Kuzmin et al., 2016 recorded in the Brazilian Neotropics (Kuzmin et al., 2016; Morais et al., 2017). In Mexico, two species have been reported infecting colubrid snakes: *S. lamothei* (Martínez-Salazar and León-Règagnon, 2006) parasite of *Leptodeira maculata* (Hallowel) in the States of Jalisco, Colima and Michoacán, on the Pacific slope of this country, and *S. fuscovenosa* (Railliet, 1899) in the States of Campeche, Guerrero, Jalisco, Michoacán, Oaxaca, Puebla, Querétaro, Veracruz, and Yucatán (Martínez-Salazar and León-Règagnon, 2006). During an ongoing project to increase the inventory of the helminth fauna associated to Mexican reptiles, we found several specimens of an undescribed species of *Serpentirhabdias* parasitising the nauyaca viper *Bothrops asper* (Garman) (Viperidae), which is described herein based on morphometric and molecular evidence.

The nauyaca viper is a poisonous snake with wide distribution from sea level up to 1,300 meters above sea level (masl), inhabiting tropical and subtropical forests of Mexico in the States of Querétaro, Hidalgo, San Luis Potosí, Tamaulipas, Puebla, Veracruz, Campeche, Tabasco, Quintana Roo, Oaxaca, Chiapas and Yucatán (Campbell et al., 2004; Saldarriaga-Cordoba et al., 2017). The snake has large body size reaching 1.50 to 2.0 meters. The new species of lungworm described herein is the first recognized species of *Serpentirhabdias* parasitising a member of Viperidae from Mexico and North America.

## Materials and Methods

A female of *B. asper* was collected in Tlatlauquitepec, Puebla, Mexico, near La Soledad Dam (19° 57′ 59.1″ N, 97° 26′ 50.9″ W; 796 masl) in August 2022. Total length (TTL; 1.34 m), snout-vent length (SVL; 1.17 m) and tail length (TL; 0.17 m) was obtained using a measuring tape (accuracy: 0.01 m); body weight (BW; 588.2g) was measured with a digital scale. The snake was found dead with a 10 cm fresh wound behind the head, inflicted with a bowie knife by local people. Host was collected under permission issued by the Secretaría de Medio Ambiente y Recursos Naturales (SGPA/DGVS/03184/22). The specimen was put inside a cooler filled with ice and transferred to the Laboratorio de Helmintología, Instituto de Biología-UNAM (IBUNAM). In the laboratory, it was dissected in order to recover the helminths following Lamothe-Argumedo (1997) and subsequently deposited in the Colección Nacional de Anfibios y Reptiles (CNAR) at IBUNAM (IBH 35835).

Lungworms were removed from the lung, counted in situ and placed in saline (0.65%). For their morphological study, some worms were fixed in 4% hot formalin and preserved in ethanol 70%; the remaining specimens were placed directly in absolute ethanol and stored at −4 °C for molecular procedures. Lungworms were cleared with 1:1 mixture of alcohol-glycerin, as a temporary mounting medium for examination under light microscopy. Measurements (mean followed by range and standard deviation) are in micrometers if no other unit is indicated. Line drawings were made with a microscope equipped with a camera lucida. Identification of worms required observation under a scanning electron microscope (SEM); therefore, some nematodes were dehydrated in a graded ethanol series and dried to critical point with CO_2_, then coated with a gold–palladium mixture and mounted on metal stubs with silver paste. Specimens were studied with a Hitachi Stereoscan Model S2469N at 15 kV at Laboratorio Nacional de Biodiversidad (LANABIO), IBUNAM. Type specimens were deposited at Colección Nacional de Helmintos (CNHE), IBUNAM.

### DNA extraction, PCR amplification and sequencing

Five nematodes previously fixed in absolute ethanol were randomly selected and put separately in 1.5 mL microcentrifuge tubes at room temperature, waiting for evaporation of the ethanol excess to be eliminated. Tissue digestion and DNA extraction were performed with EZ-10 Spin Column Genomic DNA Minipreps Kit, Bio Basic Inc. (Ontario, Canada) according to the manufacturer's instructions.

Mitochondrial cytochrome *c* oxidase subunit 1 (COI) was amplified. PCR reactions were prepared in a total volume of 15 μL containing: 2 μL of template DNA, 0.2 μL of each primer (LCO1490: 5′-GGTCAACAAATCATAAAGATATTGG -3′ and HCO2198: 5′-TAAACTTCAGGGTGACCAAAAAATCA-3′) (Folmer et al., 1994), 3 μL of 5x MyTaq Reaction Buffer and 0.1 μL MyTaq DNA Polymerase (Bioline Cat. BIO-21105), and 9.5 μL of RNAse-free H_2_O. Amplification started with an initial denaturing of 94 °C for 5 min, then 30 cycles with a first step of 94 °C for 45 s, a second step of 48 °C for 45 s and a third step of 72 °C for 1 min, and a final extension of 72 °C for 7 min. PCR reaction products were visualized by agarose gel electrophoresis and purified using CentriSep 96 filter plates (ThermoFisher Scientific) with Sephadex G-50 (Cytiva, Marlborough, Massachusetts). Sequencing reactions were prepared in a total volume of 10 μL using 0.4 μL of BigDye Terminator v. 3.1 (Applied Biosystems®, Waltham, Massachusetts), 2 μL of 5× Reaction Buffer, 4 μL of ddH_2_O, 1 μL of primer [10 μM], and 3 μL of purified PCR product. Samples were purified using Sephadex G-50 then 25 μL of 0.5 mM EDTA was added to each sample to be finally sequenced in an ABI-PRISM 3100 (Applied Biosystems®, Waltham, Massachusetts) sequencer instrument at LANABIO, IBUNAM.

### Phylogenetic analysis

For the phylogenetic analysis, we used as outgroup some sequences of *Rhabdias* species available in GenBank following Müller et al. (2018) (Table 1); we constructed a matrix with a total of 19 COI gene sequences; to align those sequences we used the online version of MAFFT v.7 (Katoh et al., 2019) with default parameters and made a final manual editing of the endpoints in Mesquite v. 3.51 (Maddison and Maddison, 2018). JModeltest v. 3.0 was used to infer the best evolution model (Anderson and Burnham, 2004). Phylogenetic analysis was performed with Maximum Likelihood (Stamatakis et al., 2005), bootstrap-based resampling with 10,000 iterations to assess nodal supports. Bayesian inference was performed with the program MrBayes v. 3.2.7 (Ronquist et al., 2012). The settings were fixed as follows: 2 simultaneous runs with 4 Markov Chains Monte Carlo (MCMC) for 10 million generations, sampling every 1000 generations, a heating parameter value of 0.2, and a “burn-in” of 25%. The convergence statistics were checked using Tracer v. 1.7 (Rambaut et al., 2018). A 50% majority-rule consensus tree representing the posterior probability distribution of clades was produced for the sampled trees**.** Trees were visualized in Fig Tree v.1.4.4 (Rambaut, 2006). Additionally, uncorrected pairwise p-distances were calculated using the program MEGAX and converted to percentage difference by multiplying the p- distance value by 100 (Stecher et al., 2020).

**Table 1:** GenBank accession numbers of sequences of *Serpentirhabdias* species included in the phylogenetic analyses, associated host, localities and references.

Species	# GenBank	Host	Localities	Reference
*Serpentirhabdias* c.f. *fuscovenosa*	MH281970	*Nerodia erythrogaster*	Tennessee, USA	Machado et al. (2018)
*Serpentirhabdias moi*	MH281968	*Chironius exoletus*	Pará, Brazil	Machado et al. (2018)
*Serpentirhabdias atroxi*	MH281969	*Bothrops atrox*	Pará, Brazil	Machado et al. (2018)
*Serpentirhabdias mussuranae*	MK681422	*Clelia clelia*	Amazon, Brazil	Kuzmin et al. (2020)
*Serpentirhabdias viperidicus*	KX354358	*Bothrops moojeni*	São Paulo, Brazil	Morais et al. (2017)
*Serpentirhabdias mexicanus* **n. sp.**	**OR583874, OR883410-13**	*Bothrops asper*	Puebla, Mexico	Present study
*Serpentirhabdias eustreptos*	MK681423	*Pantherophis obsoletus*	Nebraska, USA	Kuzmin et al. (2020)
*Serpentirhabdias fuscovenosa*	MH281972	*Natrix natrix*	Kyiv, Ukraine	Machado et al. (2018)
*Serpentirhabdias elaphe*	MK681424	*Zamenis longissimus*	Zakarpatska Oblast, Ukraine	Kuzmin et al. (2020)
*Serpentirhabdias lamothei*	KC130743	*Leptodeira* sp.	Colima, Mexico	Prosser et al. (2013)
*Rhabdias africanus*	MG428411	*Sclerophrys gutturalis*	Limpopo Province, South Africa	Kuzmin et al. (2017)
*Rhabdias bufonis*	MK681425	*Rana temporaria*	Vicinities of Kyiv, Ukraine	Kuzmin et al. (2020)
*Rhabdias pseudosphaerocephala*	MK860758	*Rhinella marina*	Nicaragua	Willkens et al. (2019)
*Rhabdias fuelleborni*	OP654198	*Rhinella marina*	Brazil	Müller et al. (2023)

Sequence obtained in the present study is in bold font.

## Results

*Serpentirhabdias mexicanus* **n. sp.** Velázquez-Brito, García-Prieto, León-Règagnon & Garduño-Montes de Oca

Phylum Nematoda Cobb, 1932

Class Chromadorea Inglis, 1983

Order Rhabditida Chitwood, 1933

Family Rhabdiasidae Railliet, 1916

Genus *Serpentirhabdias* Tkach, Kuzmin & Snyder, 2014

### Taxonomic summary

*Type host:* Nauyaca or terciopelo pit viper *Bothrops asper* (Garman) (Serpentes: Viperidae).

*Type locality:* Tlatlauquitepec, Puebla, México (19° 57′ 59.1″ N; 97° 26′ 50.9″ W).

*Site of infection:* Lung.

*Prevalence:* 100% (1 infected host out of 1 examined).

*Intensity of infection*: 29 individuals/host.

*Material deposited:* Holotype, female, Colección Nacional de Helmintos del Instituto de Biología, Universidad Nacional Autónoma de México, CNHE 11875; paratypes CNHE 11876 and 11877.

*GenBank accession:* COI sequences OR583874, OR883410, OR883411, OR883412 and OR883413.

*Etymology:* The new species is named after the country in which it was found.

### Morphological description

**Fig. 1:**
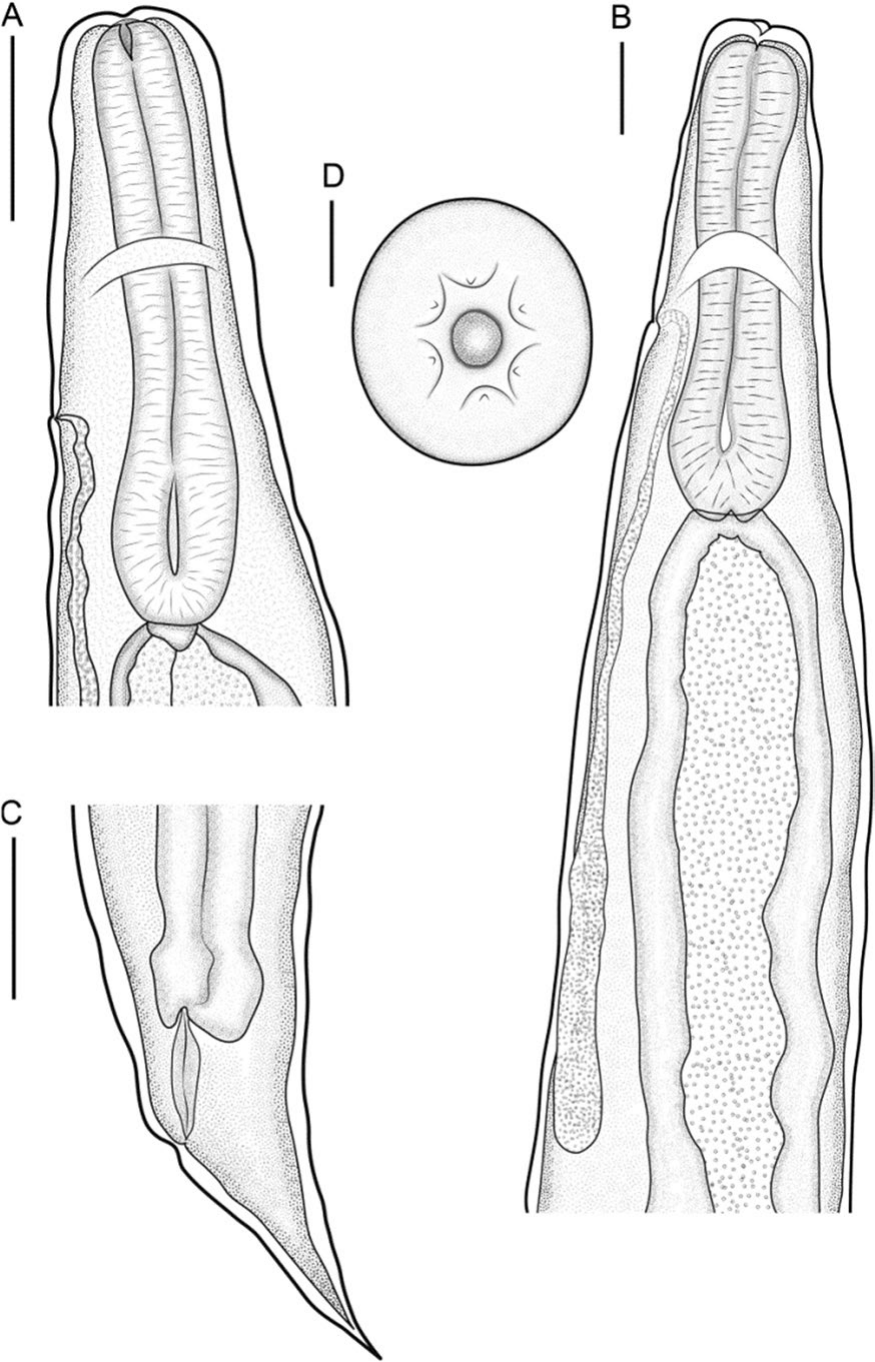
*Serpentirhabdias mexicanus* **n. sp.**, line drawings. Adult female parasite of *B. asper*. (A) Anterior region, lateral view; (B) Anterior region of body, lateral view; (C) tail, lateral view; (D) Apical view of oral region, showing papillae and lips. Scale Bar: A-100 μm, B-50 μm, C-100 μm, D-20 μm

**Fig. 2:**
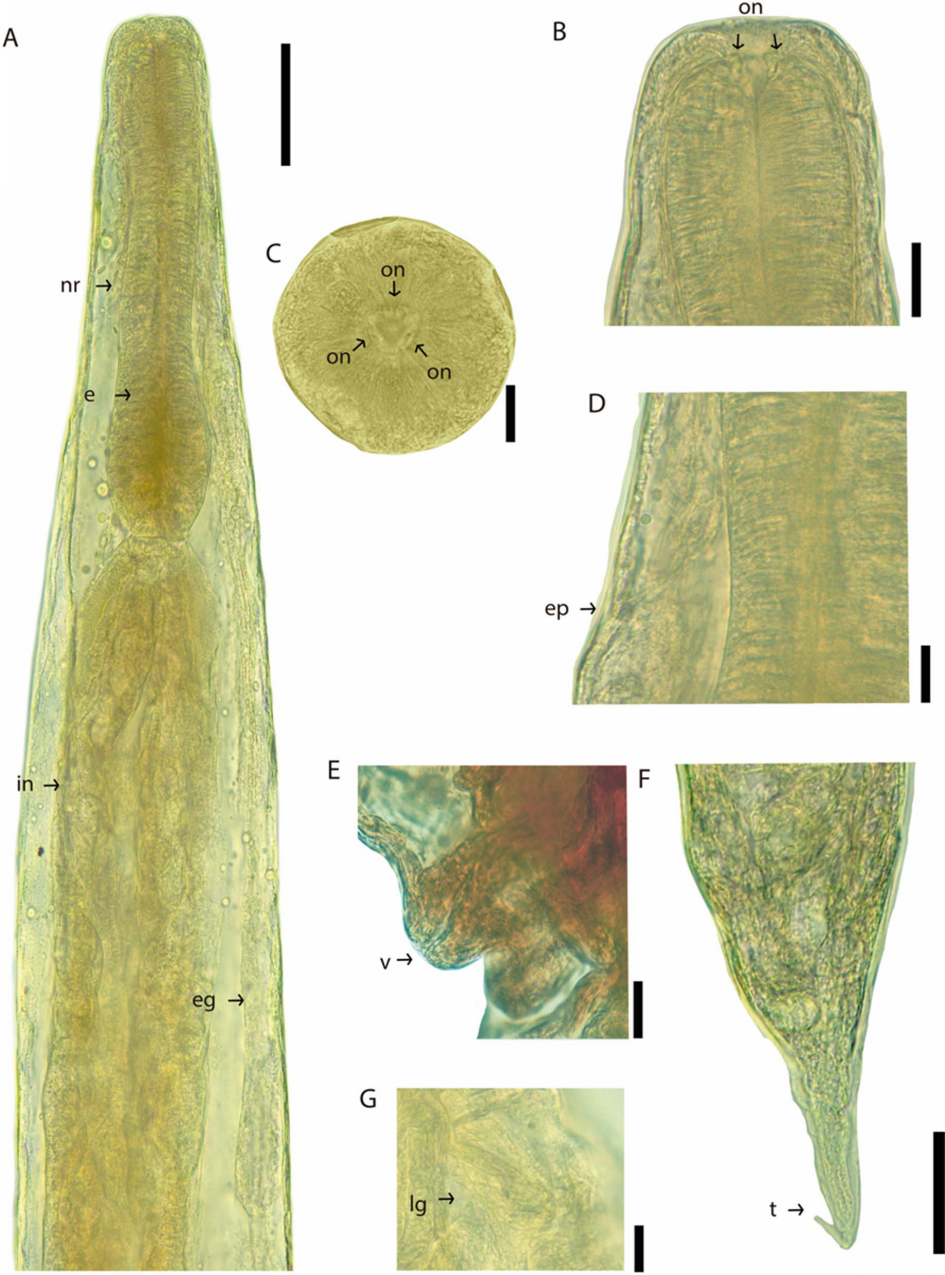
*Serpentirhabdias mexicanus* **n. sp.**, light micrographs. (A) Anterior end, [nr] nerve ring, [e] esophagus, [eg] excretory gland, [in] intestine. (B) [on] Onchia lateral view. (C) Apical view. (D) [ep] Excretory pore. (D) [v] Vulvar protruding lips. (F) [t] Tail, lateral view. (G) [lg] Larvated egg. Scale Bar 20 µm (C, B, D, E, G), 50 µm (A, F).

**Fig. 3:**
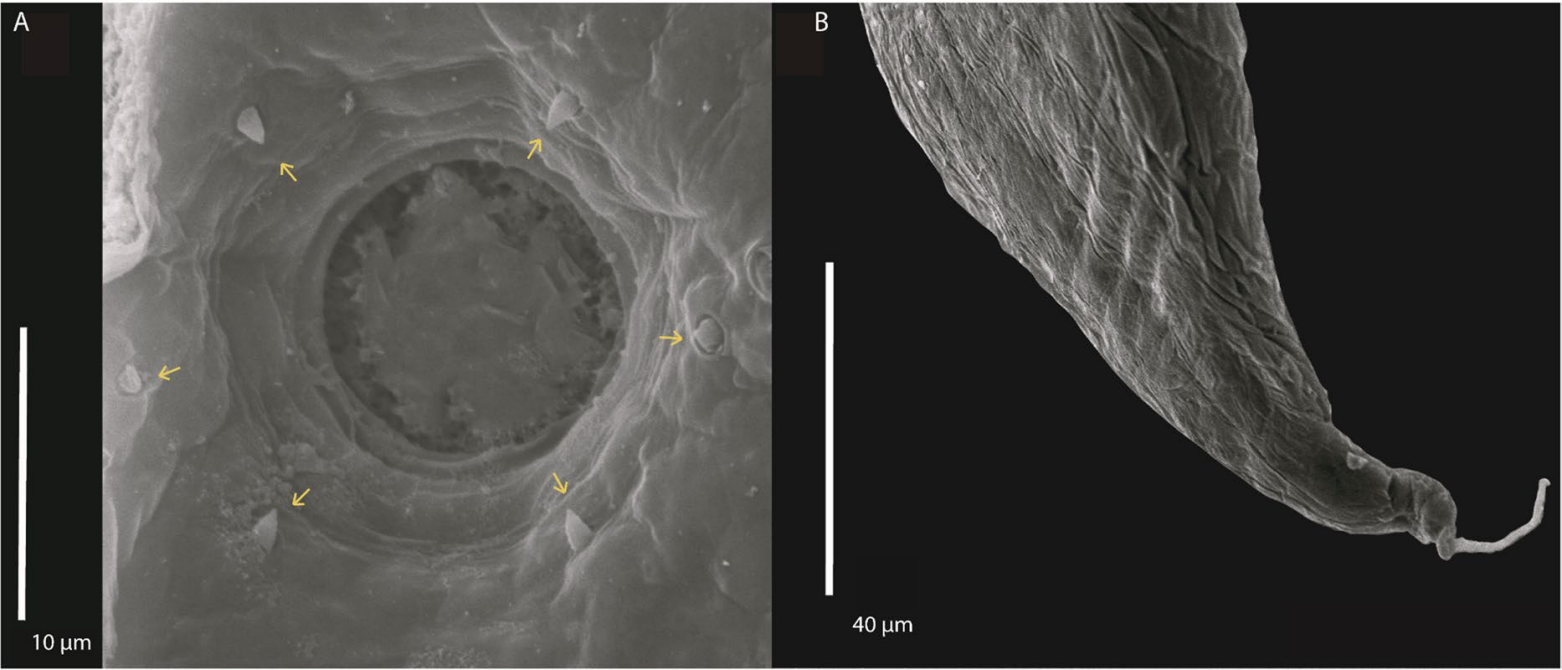
*Serpentirhabdias mexicanus* **n. sp.** Scanning electron micrographs. (A) Oral opening round and small sclerotized triangular papillae on the lips, (B) Very sharp and conical tail, involved with a layer of cuticle.

Based on ten adult females. Body 5.88 (5.247–6.361 ± 0.306) mm long. Cuticle slightly inflated at anterior [6.25 (1–13 ± 3.575) wide] and posterior ends of body (Figs. 1A, 1B, 1C, 2A and 2B). Anterior end rounded (Figs. 1D, 2C); sharp and conical posterior end (Fig. 1C). Body width 280 (227–372 ± 42.66) at vulva level; 171 (136–236 ± 35.87) at esophageal–intestinal junction. Oral opening round and wide in apical view; with six lips similar in shape and size, arranged in two lateral groups of three each; each lip with sclerotized triangular small papillae (Figs. 1D, 2C, 3A). Buccal capsule and vestibulum absent. Three pairs of small and slightly sclerotized onchia in esophagostome (Fig. 2B). Club-shaped esophagus 302 (273–330 ± 19.99) long, representing 5.15% (4.29–5.73 ± 0.47) body length; ending in a dilated bulb 79 (73–86 ± 3.80) long by 75 (64–86 ± 7.80) wide. Nerve-ring encircling esophagus, posterior to its mid-length, 168 (159–175 ± 4.94) from anterior end of body (Figs. 1A, B and 2A). Intestine brownish-black in uncleared specimens, parallel to uterus, without loops along body, running towards rectum. Excretory pore small, posterior to nerve ring, at 203 (190–215 ± 7.61) from anterior end (Figs. 1A, B, and 2D); excretory duct thin and short. Sub-ventral excretory gland relatively thin and elongated; 0.429 (0.360–0.495 ± 0.06) long by 0.045 (0.036–0.054 ± 0.007) wide, located near to esophageal bulb, ending after the esophagus-intestinal junction (Figs. 1B, 2A). Reproductive system typical of Rhabdiasidae (amphidelphic). Vulva pre-equatorial, located at 2.65 (2.33–2.99 ± 0.230) mm from anterior end, papillae absent. Vulval lips slightly protruded (Fig. 2E). Uterus with numerous eggs [137 (120–150 ± 12.58)], in different stages of development; eggs located near vulva, fully developed with visible larvae. Eggs 74 (68–86 ± 6.58) × 37 (35–44 ±2.71) (Fig. 2G). Short rectum, slightly cuticularized (Figs. 1C, 2F). Conical and pointed tail (Figs. 1C and 2F). Tail length 153 (122–170 ± 12.75), representing 2.60% (2.13–2.99 ± 0.24) of total body length.

### Remarks

*Serpentirhabdias mexicanus* **n. sp.** was assigned to this genus due to the presence of six lips similar in shape and size, arranged in two lateral groups; body cuticle thin and without conspicuous inflation, vulva pre-equatorial and buccal capsule absent, as well as by inhabiting the lung of snakes (Tkach et al., 2014; Kuzmin et al., 2016).

*Serpentirhabdias* comprises twenty species distributed worldwide (Kuzmin and Tkach, 2022); species of the genus can be separated in two groups because of the presence (four species) or absence (sixteen species) of onchia in the esophagostome. The new species belongs to the group with onchia, together with *S. moi* Machado et al., 2018, *S. mussuranae* Kuzmin et al., 2020, *S. atroxi* Kuzmin et al., 2016 and *S. viperidicus* Morais et al., 2017. These four species also lack buccal capsule as in the new species. An interesting finding among the five species having onchia is the formation of two subgroups based on the morphology of the oral opening. Species that parasitize colubrids have a triangular oral opening (*S. moi* and *S. mussuranae*), while those that parasitize viperids have a rounded mouth opening (*S. atroxi*, *S. viperidicus* and *S. mexicanus* **n. sp.**).

*Serpentirhabdias mexicanus* **n. sp.** differs from *S. viperidicus* by the absence of a short vestibulum (present in *S. viperidicus*), the length of the tail [153 (122–170) and 201 (123–208), respectively), as well as by the presence of slightly protruded vulval lips in *S. mexicanus* **n. sp.** and not protruded vulval lips in *S. viperidicus*. Furthermore, the presence of two excretory glands in *S. viperidicus* (and only one in the new species) allows the two species to be differentiated. Other traits distinguishing these two species are geographic distribution and the type host: *S. mexicanus* **n. sp.** was found in *B. asper* in central Mexico, specifically in Puebla State, while *S. viperidicus* parasitizes *B. moojeni* in Brazil (Morais et al., 2017).

*Serpentirhabdias mexicanus* **n. sp.** can be distinguished from *S. atroxi* by body length [5.38 (5.25–6.36)] mm in the new species and [4.32 (3.4–4.5) mm in *S. atroxi*], but particularly by the number of excretory glands (1 in the new species and 2 in *S. atroxi*). In addition, *S. atroxi* presents small internal and external labial papillae, while *S. mexicanus* **n. sp.** only has one external papilla on each lip. From an ecological point of view, these species differ by their geographic distribution and type host; *S. atroxi* has been found in Brazil, parasitising *B. atrox* (Kuzmin et al., 2016).

### Phylogenetic analysis

**Fig. 4:**
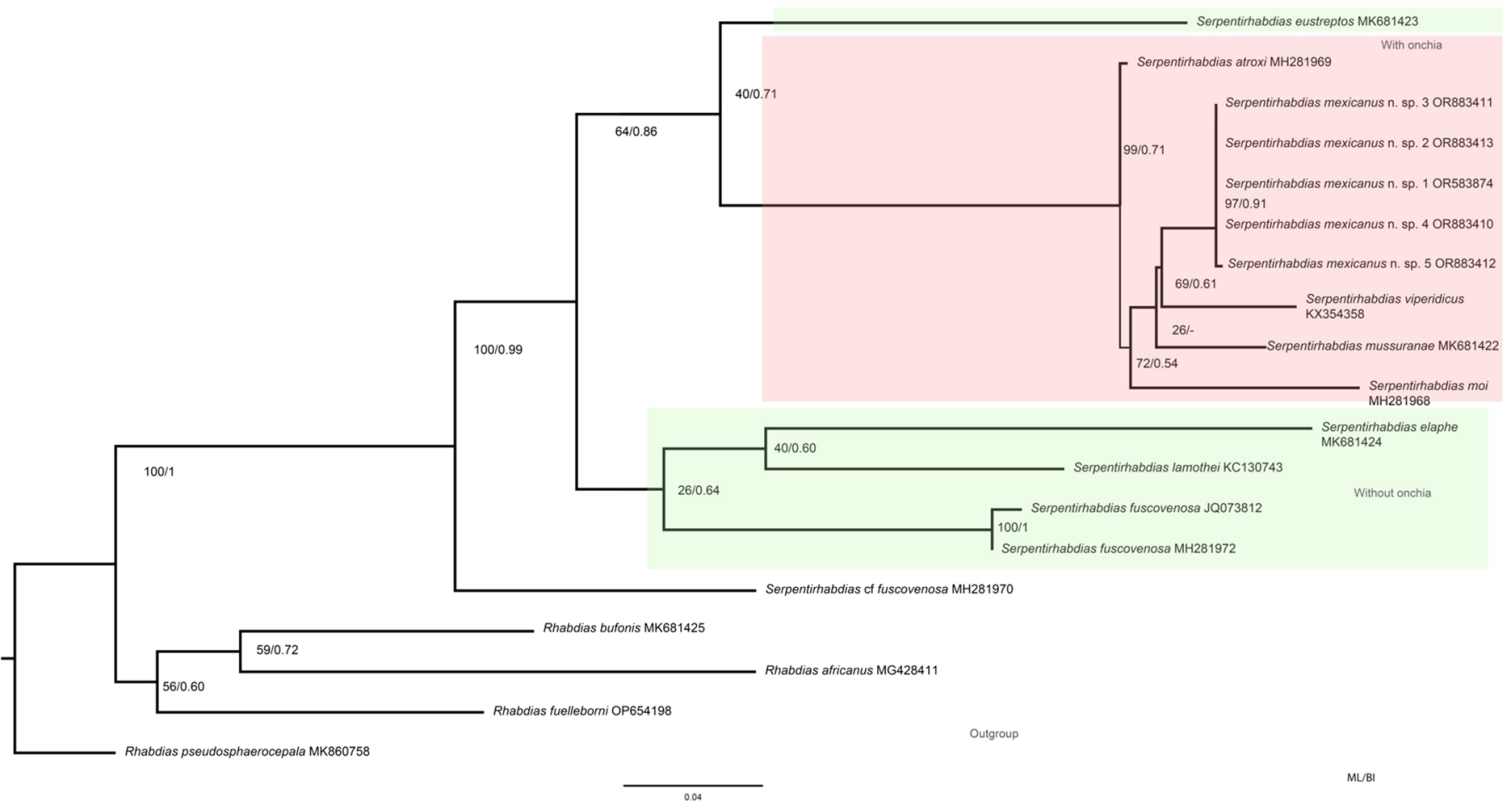
Phylogenetic tree of Maximum Likelihood and Bayesian Inference based on COI gene. Within *Serpentirhabdias*, species with onchia in the esophagostome form a monophyletic group.

In the phylogenetic hypotheses obtained based on COI sequences, using either inference method, *Serpentirhabdias* is confirmed as a monophyletic genus; sequences of individuals of the new species are grouped in a well supported monophyletic clade. Species of *Serpentirhabdias* with onchia group together, and within this group, *S. mexicanus* **n. sp.** is phylogenetically closest to *S. viperidicus*, while *S. mussuranae* is related to *S. moi* (Fig. 4). According to the percentage genetic distance matrix presented in Table 2, the new species differs from the rest of its congeners in an interval that ranges between 3% (*S. atroxi*) and 16% (*S. elaphe*). Genetic divergence among sequences of 5 specimens of *S. mexicanus* was 0%.

**Table 2:** Genetic distances (expressed as percentage difference) between the partial nucleotide sequences of the COI gene of the *Serpentirhabdias* species included in this study

*Serpentirhabdias* species	*S. fuscovenosa*	*S. viperidicus*	*S. elaphe*	*S. moi*	*S. eustreptos*	*S.* cf. *fuscovenosa*	*S. mussuranae*	*S. atroxi*	*S. mexicanus* **n. sp.**
*S. fuscovenosa*	0								
*S. viperidicus*	13	0							
*S. elaphe*	13	16	0						
*S. moi*	12	9	17	0					
*S. eustreptos*	12	14	14	14	0				
*S.* cf. *fuscovenosa*	11	13	14	13	14	0			
*S. mussuranae*	13	6	16	8	14	13	0		
*S. atroxi*	12	4	15	6	13	12	4	0	
*S. mexicanus* **n. sp.**	13	5	16	7	13	13	4	3	0

## Discussion

*Serpentirhabdias mexicanus* **n. sp.** is the third species of the genus recorded in Mexico along with *S. lamothei* and *S. fuscovenosa* (see Martínez-Salazar and León-Règagnon, 2006). However, it is the first species with onchia described in the country (see Kuzmin et al., 2020). Among *Serpentirhabdias* species, onchia were first described in *S. atroxi*. However, these structures have also been described in species of *Entomelas* and *Rhabdias*, and according to Kuzmin et al. (2016), this trait represents an evolutive convergence for these three genera.

Phylogenetic relationships within Rhabdiasidae are not well resolved, mainly due to the lack of molecular information. However, the clades recovered in the present analysis with COI sequences available in Genbank to date (Fig. 4), corroborated the monophyly of *Serpentirhabdias* as proposed by Morais et al. (2017), based on a phylogenetic analysis of ribosomal DNA. The present phylogenetic analysis of COI sequences, group species of *Serpentirhabdias* with onchia in a monophyletic clade, although Bayesian support was low. On the other hand, the assemblage of species according to the shape of oral opening and host family [round in species parasitising Viperidae (*S. atroxi*, *S. viperidicus* and *S. mexicanus* **n. sp.**)] and triangular in Colubridae (*S. moi* and *S. mussuranae*), was not supported. We consider that more solid conclusions on the relationships between the morphology of oral opening and host family of *Serpentirhabdias* spp. (Viperidae-Colubridae) will require a broader sampling effort, including wide geographic and host spectrum. It is worth noting that species with onchia have been described solely in the American continent, specifically in Brazil and Mexico (Kuzmin et al., 2016, 2021; Morais et al., 2017; Machado et al., 2018; present study). Erection of *Serpentirhabdias mexicanus* **n. sp.** as a new species is supported by morphological, molecular, ecological (geographic distribution and host) and phylogenetic evidence.

## Data Availability

The sequences generated and analyzed in this study have been deposited in the GenBank database under the accession numbers: OR583874, OR883410-13. Not applicable.
